# Abomasal Disorders in Dairy Cows During the Transition Period: Interconnected Risk Factors and Repercussions on Health and Culling Rate

**DOI:** 10.3390/ani16172706

**Published:** 2026-09-01

**Authors:** Enzo Freire Santana do Amaral, Gabriela Anteveli, João Pedro Matiello, Barbara Andrade Alves, Guilherme Silva Lemos, Helena Ferreira Lage, Rodrigo Melo Meneses, Tiago Facury Moreira, Elias Jorge Facury Filho

**Affiliations:** 1 Department of Veterinary Clinical and Surgical Sciences, School of Veterinary Medicine, Federal University of Minas Gerais, Belo Horizonte 31270-901, Minas Gerais, Brazil; enzosantanavet@gmail.com (E.F.S.d.A.); gabianteveli21@gmail.com (G.A.); guilhermelemos.vet@gmail.com (G.S.L.); menesesrm@gmail.com (R.M.M.); eliasfacury@gmail.com (E.J.F.F.); 2 Embrapa Goats and Sheep, Sobral 62010-970, Ceará, Brazil; jp.mtll@gmail.com; 3 Department of Veterinary Medicine, Federal University of Lavras, Lavras 37200-900, Minas Gerais, Brazil; andradealves.barbara@gmail.com; 4 Department of Animal Science, School of Veterinary Medicine, Federal University of Minas Gerais, Belo Horizonte 31270-901, Minas Gerais, Brazil; helenaf.lage@gmail.com

**Keywords:** abomasal displacement, abomasal ulcers, dairy cattle, transition period, immunometabolism, culling

## Abstract

The transition period in dairy cows increases susceptibility to various diseases. Abomasal diseases, particularly abomasal displacement (AD) and abomasal ulcers (AU), are important causes of production losses, early culling, and compromised animal welfare. However, few contemporary reviews have comprehensively synthesized these conditions. This narrative review aims to critically synthesize the main risk factors, integrate the underlying pathophysiological mechanisms, and present recent advances in diagnosis and treatment. Evidence indicates that AD and AU are disorders closely associated with a negative energy balance, hypocalcemia, and systemic inflammation. These factors interact through complex immunometabolic pathways. Inflammatory activation, often triggered by endotoxins or sterile inflammation, increases pro-inflammatory cytokine production, disrupts calcium homeostasis, and gastrointestinal neuromuscular function. Consequently, there is a reduction in ruminal and abomasal motility, decreased dry matter intake, and a greater predisposition to gas accumulation and abomasal displacement, in addition to alterations in the integrity of the abomasal mucosa that favor ulcer formation. Furthermore, concomitant diseases such as subclinical ketosis, metritis, lameness, and subacute ruminal acidosis amplify these processes. Despite advances in diagnosis, clinical overlap with other digestive disorders remains a challenge. Preventive strategies should prioritize nutritional management, modulation of inflammation, and maintenance of metabolic homeostasis. Current evidence suggests that AD and AU may be associated with systemic immunometabolic dysregulation rather than representing entirely isolated disorders. However, further studies are needed to confirm the proposed mechanisms in cattle and to determine their implications for prevention and control strategies.

## Simple Summary

The transition period is marked by several challenges to the health and productivity of dairy cows. Abomasal disorders such as abomasum displacement (AD) and abomasal ulcers (AU) result in compromised animal welfare and increased culling rates. This review summarizes the pathophysiology and the interconnection between risk factors, diagnosis, and treatment of AD and AU. Evidence indicates that these disorders are strongly linked to negative energy balance, ketosis, hypocalcemia, systemic inflammation, and concurrent diseases such as metritis, lameness, and subacute ruminal acidosis. These conditions interact through complex immunometabolic pathways that reduce feed intake, impair ruminal and abomasal motility, and increase the likelihood of gas accumulation and displacement. In addition, prolonged exposure of the abomasal mucosa to acidic contents and inflammatory mediators can favor ulcer formation. Although current evidence is largely associative, it supports the hypothesis that systemic immunometabolic dysregulation may play a central role in the pathogenesis of both AD and AU.

## 1. Introduction

The transition period is one of the most physiologically challenging stages in dairy cows’ lives. During this phase, homeorhetic and homeostatic adaptations are required to support late fetal growth, colostrogenesis, and the onset of lactation [1]. Historically, the transition period has been defined as the 3 weeks before and 3 weeks after calving, and transition cow disorders were primarily attributed to negative energy balance (NEB), immune suppression, inflammation and maladaptive endocrine adaptations [2].

However, our knowledge of the physiology and metabolism of dairy cows during the transition period has advanced substantially over the past two decades, particularly regarding the role of inflammatory processes in metabolic adaptation and disease susceptibility [3,4]. In this context, inflammation is increasingly recognized not only as a potential contributor to disease but also as an essential component of the physiological adaptations required for successful transition [5]. Sterile inflammatory responses may be triggered by catecholamines released through sympathetic nervous system activation, as well as by glucocorticoids released via the hypothalamic–pituitary–adrenal axis during peripartum stress, representing additional signals capable of modulating inflammatory processes [3].

Accordingly, the reduction in dry matter intake (DMI) observed during this period likely reflects the combined influence of inflammatory signaling and coordinated homeorhetic adaptations, which may also occur in clinically healthy, high-producing cows [1,3]. Therefore, adverse health outcomes in transition dairy cows appear to be more closely associated with the degree of activation and regulation of inflammatory responses, rather than with their mere presence, with dysregulation potentially representing a key factor in the progression toward disease [3,5].

During this period, the susceptibility to metabolic disorders and the risk of culling increase considerably [6,7]. Accordingly, conditions such as hypocalcemia, fatty liver, ketosis, retained placenta, metritis, and mastitis affect animals individually or simultaneously, with varying prevalence [8,9]. Mastitis, reproductive problems, and injuries to the locomotor system are considered the main causes of culling in dairy herds [9]. Increased early culling results in compromised farm profitability, increased environmental damage associated with the activity, and a negative impact on consumer perceptions [9].

Among the disorders affecting transition cows, digestive diseases deserve particular attention because they are closely linked to metabolic disturbances, inflammatory activation, impaired feed intake, and reduced productive performance [10,11,12,13]. Similarly, other forestomach conditions (i.e., ruminal distension, dysmotility disorders, and acute abdomen syndrome) become more frequent during periods of dietary transition and changes in nutritional management [14,15]. The occurrence of these conditions may exacerbate inflammation and metabolic stress [4,5].

Abomasum displacement (AD) has been reported in dairy cows since the 1950s and remains prevalent during the transition period [16,17,18], significantly contributing to increased culling rates [6]. Abomasal ulcers (AU) are characterized by lesions of varying severity in the mucosa and other histological layers of the abomasum. In more severe cases, the condition can perforate and culminate in peritonitis, leading to severe pain in the animals, as well as mortality [19].

The estimated economic loss associated with each case of AD is approximately US$700 [20], reflecting both direct costs, such as diagnosis and treatment, and indirect losses related to reduced productivity and premature culling. In addition, AD has been associated with a reduced likelihood of pregnancy at first service by 120 days in milk, decreased milk and milk-solids yields, and an increased risk of culling [21]. In contrast, studies evaluating the productive and reproductive consequences of AU are scarce. Nielsen et al. [22] investigated the effects of non-perforating abomasal ulcers in dairy cattle and reported that mild lesions (Type I ulcers) and abomasal nodules were not associated with reductions in milk yield, carcass weight, or reproductive performance. However, the effects of more severe forms of AU on productivity, reproduction, and longevity remain poorly understood and warrant further investigation.

AD and AU have multifactorial etiologies [23,24] and pose challenges for diagnosis and treatment [13,25]. Therefore, this comprehensive review aims to associate the main risk factors and their mechanistic links with abomasal disorders in transition dairy cows, providing recent advances in the diagnosis and treatment of abomasal displacement and ulcer cases.

## 2. Review Methodology

This narrative review synthesizes and evaluates current evidence regarding the role of systemic immunometabolic dysregulation in the pathogenesis of abomasal displacement and ulcers.

A literature search was conducted between October 2025 and March 2026 using Google Scholar, PubMed, Scopus, and Web of Science databases, encompassing studies published between 1990 and 2026. The search strategy included combinations of the following keywords: “abomasal displacement,” “abomasal ulcers,” “transition period,” “dairy cows,” “negative energy balance,” “epidemiology,” “risk factors,” “hypocalcemia,” “ketosis,” “inflammation,” and “immunometabolism.” Greater emphasis was placed on studies published within the last 15 years to support the main sections of the review, while seminal studies published before 1990 were retained when considered essential for historical context and for the development of current concepts.

Titles and abstracts were initially screened for relevance, followed by full-text evaluation. Approximately 128 publications were ultimately included and cited throughout the review. Because the purpose of this study was to provide a broad narrative overview rather than a systematic assessment, a formal study selection flow diagram was not applied.

Eligibility was determined according to the relevance of each study to the epidemiology, pathophysiology, risk factors, diagnosis, treatment, and immunometabolic mechanisms associated with abomasal disorders. Original research articles, review articles, and case reports were considered for inclusion.

Studies involving other animal categories, ruminant species, or even human models were included when they provided mechanistic insights relevant to abomasal disorders. The exclusion criteria were studies unrelated to abomasal disorders or with unclear methodologies.

## 3. Abomasal Displacement

In non-pregnant cows, the abomasum is located from the xiphoid region to the 9th or 10th right intercostal space in a caudoventral position to the right of the rumen [26]. The prevalence of AD has been reported in several studies worldwide, ranging from 0.26 to 7% [16,18,23,27,28]. However, the incidence rate increases considerably during the transition period, with herd-level prevalence considered acceptable below 3% and problematic above 6% [20,23]. Left displaced abomasum (LDA) is the most common [13,16,18]. Nevertheless, right displaced abomasum (RDA) and abomasal volvulus (AV) present more challenging consequences and, when not treated adequately, result in clinical signs with more serious repercussions and increased mortality rates [16].

Although the pathogenesis of AD has not been fully elucidated, it is believed that conditions that promote gas accumulation and impaired abomasal motility or stasis of the organ predispose to AD or AV [17]. One study investigating the pathogenesis of AD was conducted by González-Martin et al. [29]. They concluded that the abomasum assumes different positions, being able to displace caudally, laterally, cranially, and medially, as well as laterally to the omasum. When the abomasum is displaced cranially and laterally from the omasum, intraluminal pressure increases, making it more susceptible to AV. Clinical aspects of AD and AV will be discussed in more detail below.

### 3.1. Risk Factors

#### 3.1.1. Ketosis

**Figure 1:**
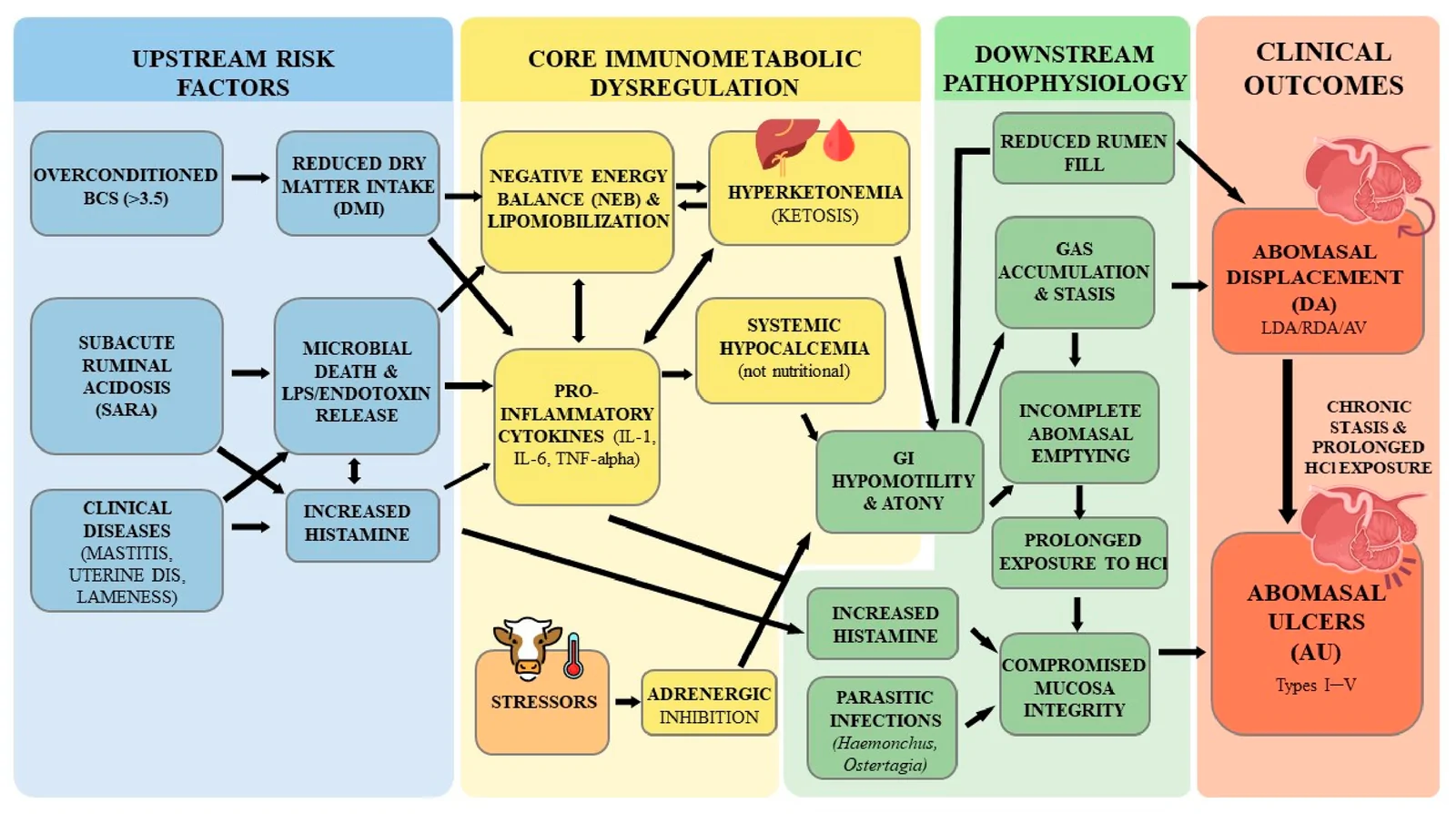
Interconnection of the main metabolic risk factors for abomasal displacement and ulcers. Overconditioned cows experience a more pronounced negative energy balance (NEB) postpartum. Reduced dry matter intake (DMI) associated with increased metabolic stress increases energy requirements. As a strategy, cows increase the production of ketone bodies capable of inducing hypomotility and abomasal atony. Transitional diseases (acidosis, mastitis, metritis, ketosis), related or not to Lipopolysaccharide (LPS-producing infectious agents, are associated with the release of inflammatory mediators. Current evidence suggests that transient hypocalcemia may represent part of an adaptive response aimed at modulating inflammatory activation during the transition period. Nevertheless, prolonged or more severe hypocalcemia can impair gastrointestinal smooth muscle function, resulting in hypomotility and potentially predisposing cows to abomasal displacement. Excessive inflammation accentuates NEB and the reduction in DMI, resulting in decreased ruminal motility and increased gas production in the rumen and abomasum. Stress factors release adrenergic mediators that cause hypomotility. Histamine produced in the rumen during acidosis may contribute to increased hydrochloric acid secretion. Hypomotility, with increased hydrochloric acid for prolonged periods, exceeds the protective capacity of the abomasal mucosa, leading to abomasal ulcers. Source: Figure 1 was created and supervised by the authors with the aid of Gemini (Google DeepMind, 2026 version 3.5 Flash).

The associations between the consequences and causality of transition disorders should be interpreted with caution [3] (Figure 1). Ketosis is characterized by the concentration of ketone bodies in the blood to levels equal to or greater than 1.2 mmol/L, although this cutoff point deserves consideration depending on the lactation stage [30].

A recent review suggests that the timing of hyperketonemia onset may influence its biological and productive implications [30]. Based on evidence from previous studies, cows that develop hyperketonemia after the first nine days of lactation often exhibit greater milk production, suggesting that increased ketone body concentrations may, in some cases, reflect a physiological adaptation to meet the energetic demands of lactation during a prolonged NEB. In contrast, hyperketonemia occurring within the first nine days postpartum has been consistently associated with impaired productive performance and an increased risk of health disorders. These observations highlight that elevated ketone body concentrations may reflect either a physiological adaptation to support milk production or a pathological state associated with impaired health, depending on the metabolic context.

Clinical signs of ketosis include reduced appetite, altered feeding patterns, reduced milk production, and neurological signs in severe cases [31]. Reduced DMI is a characteristic of cows that develop ketosis [32]. Consequently, cows experience a more severe NEB, which imposes greater mobilization of body reserves, such as adipose and muscle tissues, to meet energy needs [1]. During lipomobilization for the production of ketone bodies, many pro-inflammatory cytokines are released [33], and upon reaching this inflammatory status, DMI is further reduced [34]. Inflammation also impairs ruminal function and triggers reduced motility through regulatory mechanisms involving higher centers in the brain [14,34]. Consequently, reduced ruminal outflow reaches the abomasum, and the ruminal emptying rate decreases, allowing the abomasum to accumulate gas and reduce motility, predisposing it to displacement [17,20].

Hyperketonemia, both prepartum and in the immediate postpartum period, was identified as the main risk factor for AD in the study by LeBlanc et al. [16]. Yong et al. [28] conducted a five-year retrospective study of LDA cases on a farm in China. These authors demonstrated that ketosis, individually or associated with other diseases, was the metabolic disease most associated with LDA, affecting approximately 20% of the animals.

Interestingly, in cows undergoing surgical intervention to correct LDA, higher concentrations of beta-hydroxybutyrate at the time of surgery have been associated with a lower risk of culling from the herd [35]. However, Reynen et al. [35] did not consider important factors associated with herd survival rate. For example, the authors did not monitor the reproductive performance of cows after LDA correction. Reproductive performance has important effects on culling [9,36].

One possible interpretation is that ketone body concentrations at the time of diagnosis may reflect differences in metabolic status and disease severity, rather than exerting a direct causal effect on outcomes. In this context, hyperketonemia may represent a concurrent metabolic state associated with energy mobilization and disease adaptation in affected cows, which could partly explain its association with improved survival in some clinical scenarios. Accordingly, the relationship between ketosis and culling following LDA correction appears to be context-dependent and not fully elucidated [18,30,35].

#### 3.1.2. Hypocalcemia

Hypocalcemia is another prevalent condition in dairy herds, affecting 2.5–5.0% of animals in its clinical form and 25–50% of primiparous and multiparous cows in its subclinical form [37]. Cows with a total Ca concentration of 2.2 mmol/L are considered hypocalcemic, and Ca values < 1.5 mmol/L are often observed in more severe cases of clinical hypocalcemia [37,38]. Cut-off points should be evaluated cautiously, as there are discrepancies in the literature and cows may present different clinical signs for the same cut-off point. For example, Mann et al. [31], reported that some cows with tCa 1.5 mmol/L become recumbent while others do not. Reports like this reinforce the importance of the clinical-epidemiological association and of interpreting results to improve decision-making.

Recent evidence has demonstrated that calcium concentrations fluctuate dynamically during the transition period [37,39]. In more severe cases, subclinical hypocalcemia can lead to reduced milk production, impaired immunometabolism, and increased susceptibility to other transitional diseases [3,37,38,39]. However, despite recent advances in understanding postpartum calcium dynamics and their associations with milk production, health outcomes, and survival in dairy cows [39], further research is needed to determine whether these calcium fluctuations directly influence the incidence of AD.

Concomitant hypocalcemia is frequently observed in LDA-affected cows [13], and postpartum hypocalcemia was associated with a 3.1-fold increase in AD cases [40]. The association between hypocalcemia at AD diagnosis and an increased likelihood of culling is not universally supported [41], despite being reported in earlier investigations [38,39]. These conflicting results may arise from the multifactorial etiology of AD; thus, the contribution of this condition to the increased culling rate should be interpreted in conjunction with other challenges of transition and calcium metabolism, such as the resumption of reproductive activity.

However, the association between hypocalcemia and AD is strongly supported by the essential role of calcium in gastrointestinal smooth muscle contractility [42]. Consequently, systemic calcium imbalances appear to be a critical risk factor for AD, leading to tissue hypomotility and subsequent organ atony [42,43]. It has been observed that cows diagnosed with hypocalcemia show a reduction in DMI and rumination time when compared to eucalcemic cows [37,44].

#### 3.1.3. Uterine Diseases

In addition to metabolic disturbances, inflammatory reproductive diseases appear to play a relevant role in AD pathogenesis. Uterine diseases, such as retained placenta, metritis, and endometritis, are also among the most common adverse health events in transition cows [5,45].

Many uterine disorders are associated with bacterial infection by Gram-negative bacteria that cause an intense inflammatory response in the uterine lumen and, in some cases, in the systemic circulation [8,45,46]. The release of endotoxins in the form of lipopolysaccharides (LPS) has been identified as a direct inhibitory factor of abomasal motility [46]. Moreover, upon reaching an endotoxemic state, animals may develop a transient hypocalcemic state as a defense mechanism against the endotoxemia, which has been hypothesized to modulate inflammatory responses [47].

In studies that induced inflammation with intravenous infusion of LPS or in cases of sepsis, it was demonstrated that the eucalcemic state has been associated with greater inflammatory responses, culminating in more severe repercussions for the organism [47,48]. Alterations in extracellular calcium availability have been suggested to influence immune cell activation and cytokine release [46,47,48]. The macrophage activation pathways involved in endotoxin clearance release a storm of pro-inflammatory cytokines (IL-1, IL-6, TNF alpha) [46,47]. These cytokines cause more deleterious effects and are capable of inhibiting ruminal motility and decreasing DMI [34]. A significant limitation of the study by Horst et al. [47] is that it was conducted with mid-lactation cows. In the early postpartum period, the immune system is influenced by homeostatic and homeorhetic changes, which can alter its functionality and performance due to other conditions (such as hypocalcemia) [3,4,38].

#### 3.1.4. Gestational and Parturition Conditions

AD has a stronger association with other transition diseases than with mechanical causes [16,23,49], which explains the possibility of other categories (i.e., bulls, calves, and non-pregnant cows) developing the condition. Twin pregnancies appear to predispose cows to AD due to the space generated between the rumen and pregnant uterus [50]. During late gestation, adaptive anatomical changes in cows can lead to increased abdominal depth, dorsal displacement of the rumen, and vertical distance between the abomasum and duodenum [51]. Furthermore, there may be slight compression by the uterus in the most distal portion of the abomasum, which can also compromise motility, generating greater susceptibility to AD [52].

In a study with six herds in Iran conducted by Asgari et al. [18], abnormalities during calving (stillbirth, twin births, or dystocia) and prolonged gestation were associated with a 1.31-fold greater risk of developing AD. Dystocia was identified as the main factor associated with increased probability of culling AD-affected cows in the first 60 days after treatment [35]. Higher concentrations of magnesium and beta-hydroxybutyrate, as well as the control and maintenance of production, were identified as factors that maintain cows in the herd after AD treatment.

#### 3.1.5. Subacute Ruminal Acidosis

There are multiple definitions of subacute acidosis (SARA) in the literature [10,53]. In general, when ruminal fermentation exceeds the rumen’s buffering and absorptive capacity of the volatile fatty acids (VFAs) produced, there is an accumulation of these fatty acids, with a resulting drop in pH that can cause alterations in ruminal symbiosis and promote subacute ruminal acidosis [10].

The development of SARA is strongly associated with the consumption of high-concentrate diets, particularly those rich in rapidly fermentable starch sources [10,54]. The rapid fermentation of starch promotes increased production of VFAs, especially propionate, leading to a marked decline in ruminal pH. Consequently, the reduction in pH is often more pronounced and prolonged, favoring substantial alterations in the composition and function of the ruminal microbiota. In addition, diets deficient in physically effective fiber reduce chewing and rumination activity, thereby decreasing saliva secretion and the delivery of buffering compounds to the rumen. This reduction in ruminal buffering capacity impairs the neutralization of hydrogen ions (H+), further exacerbating the decline in ruminal pH and increasing the risk of SARA development [7,53,54].

Evidence suggests that a single-point measurement of ruminal pH may not accurately reflect the epidemiological status of SARA within dairy herds [53]. Rather than isolated pH values, the duration for which ruminal pH remains below critical thresholds appears to be a more reliable indicator of SARA prevalence and its associated consequences, including reduced milk fat concentration, liver abscesses, ruminal parakeratosis, and dysbiosis of the ruminal microbiota [53,54]. Recent reviews and meta-analytical evidence suggest that cows are at increased risk of developing SARA when ruminal pH remains below 5.8 for approximately 5.24 h/day or below 5.6 for at least 3 h/day [53,54].

When VFAs accumulate in the rumen, they may be transported to the abomasum, where they contribute to a reduction in abomasal pH and promote intraluminal gas accumulation [55]. Experimental evidence has demonstrated that intraluminal infusion of VFAs, particularly butyrate and propionate, into the abomasum of sheep significantly impairs abomasal motility and delays gastric emptying, as evidenced by reduced smooth muscle electrical activity [56]. Furthermore, during episodes of ruminal acidosis, microbial lysis leads to the release of LPS, which can trigger systemic inflammatory responses and further impair gastrointestinal motility, thereby contributing to abomasal dysfunction [46].

#### 3.1.6. Management and Herd Characteristics

High-producing Holstein cows appear to be at greater risk of developing AD during the transition period. Evidence suggests that the heritability of AD ranges from 0.03 to 0.53, indicating that genetic factors may contribute to disease susceptibility [57]. Furthermore, parity may influence the occurrence of AD, as multiparous cows have a higher incidence of transition disorders, including hypocalcemia, which has been associated with an increased risk of AD [37,40].

Some herd characteristics and management practices can also predispose animals to AD. For example, overconditioned cows (Body condition score-BCS greater than 3.5) during the transition experience a greater reduction in DMI and more pronounced effects of NEB than cows that maintain a moderate body condition score (3.0–3.25) [58]. Obese animals may be more susceptible to developing ketosis and other adverse postpartum conditions (e.g., retained placenta, hypocalcemia, and metritis) associated with AD [30,32]. The reduction in DMI is partially explained by the intensification of the systemic inflammatory response due to lipomobilization [33,34].

Feed management is important during this period because, when well conducted, it can prevent the occurrence of overconditioned cows throughout lactation, as well as avoid abrupt changes in feeding by adopting an appropriate adaptation period (through the gradual inclusion of starch postpartum) during the transition, thereby reducing the risk of ruminal acidosis [10,11,55].

#### 3.1.7. Lameness

Although little explored, some studies have suggested a correlation between AD and lameness [59,60,61]. Foot lesions increase considerably during the transition period and are correlated with other transition diseases. These lesions have a multifactorial etiology with risk factor-related metabolic-nutritional disorders, hormonal changes, abrupt changes in diet, and increased infection pressure [62]. According to Tschoner et al. [61], the relationship between AD and foot lesions could be associated with the prolonged time that animals with foot lesions spend in recumbency due to reluctance to remain standing, which could facilitate AD. Moreover, these results have not been confirmed in controlled studies. The same authors also demonstrated that cows with foot lesions have higher concentrations of inflammatory markers, which could indirectly contribute to AD development. Pain resulting from discomfort when bearing weight on the limb or even from the inflammatory process itself can trigger reduced ruminal motility [14] and lead to AD. Rohn et al. [59] reported that animals with some degree of lameness had a lower cure rate after surgical treatment to correct AD and a consequent increase in the likelihood of culling when compared to non-lame animals.

### 3.2. Diagnosis

Exploratory laparotomy remains the gold standard for definitive diagnosis of AD and AV [13]. However, clinical examination has been the most common method for diagnosing AD, being more accurate in diagnosing LDA than RDA and AV [13,63]. The differentiation between RDA and AV can be made through necropsy or laparotomy [13]. Braun et al. [10] and others [27,64,65,66] have extensively detailed the main clinical findings in cows with AD or AV (see Supplementary Table S1). During clinical examination, a “metallic ping” sound may be observed on auscultation, and in some cases, splashing during simultaneous auscultation of the sphincter [13]. Metallic pings are produced due to a liquid–gas interface being formed [64] and are not pathognomonic of AD [49]. They may represent other differential diagnoses (e.g., empty rumen, cecal dilation, and intestinal loop dilation) [67]. It is necessary to use methods such as ultrasound to differentiate the abomasum from other structures (such as the cecum, rumen, and intestinal loops).

In cows with LDA, the abomasum can be seen occupying the 9th to 12th left intercostal spaces. Furthermore, the pyloric canal can be detected in the left ventral region of the abdomen as a loop with a hypoechoic wall and echogenic luminal content in the cross-section [68]. RDA or AV-affected cows, ultrasonographic differentiation remains limited, but the abomasum can be seen occupying the region of the 10th–12th right intercostal spaces and, in some cases, occupying the region of the 9th intercostal space, with the dorsal and ventral margins of the abomasum located more dorsally, containing a gas cap demarcated by reverberation artifacts [69].

The metabolic alterations resulting from LDA, RDA, and AV have been well demarcated in recent studies [13,64,65] (see Supplementary Table S1). Urea was identified as the best prognostic indicator in the clinical biochemistry of animals with RDA or AV, precisely because it is associated with the animals’ dehydration status, which in turn affects other clinical parameters (such as increased heart rate) and hematological parameters, such as increased lactate and inorganic phosphorus levels and hyponatremia [65]. Animals with RDA and AV tend to present with metabolic alkalosis associated with hypochloremia and hypokalemia. A more striking characteristic of AV is the episodes of pain due to abomasal torsion, which characterizes acute abdomen syndrome [15]. However, in more advanced cases, the hemodynamic status may be disturbed, with metabolic acidosis and apathy [13,15]. Therefore, it is worth mentioning that the evolution of clinical signs in this case may alter the initial conditions and indicate other differential diagnoses.

Precision livestock technologies and artificial intelligence represent promising tools for the diagnosis and monitoring of AD [69]. Rumination sensors and accelerometers can continuously monitor behavioral and physiological changes associated with AD, including reduced rumination time, decreased activity, increased lying time, and declines in milk production, often enabling the early identification of affected cows [70,71]. In addition, machine learning prediction models that integrate data from wearable sensors, milking systems, and other herd management records have shown promise for identifying cows at increased risk of developing AD before the onset of overt clinical signs. In addition, sensor-based approaches may assist in evaluating therapeutic responses during the post-surgical period, contributing to a more accurate assessment of recovery and ruminal function following different corrective techniques [72,73]. The use of sensors and precision technologies assists in the overall monitoring of health, but does not replace a thorough clinical examination for the identification and differentiation of diseases.

Recently, AD patterns have been investigated in other associations with the overall health profile of cows, mainly the microbiome [74,75]. By comparing healthy cows and cows undergoing LDA correction treatment (seven days after surgical correction) with cows with LDA, Qin et al. [74] demonstrated that cows diagnosed with LDA had a lower relative proportion of the *Firmicutes* phylum and an increase in the *Bacteroidota* in the rumen. Interestingly, after treatment, LDA-affected cows showed bacterial diversity and metabolism similar to those of healthy cows. Advanced omics technologies have significant limitations owing to their cost. Hence, their use in research contributes to understanding the pathogenesis of AD; nevertheless, their diagnostic value at the farm level remains a challenge. Discussing the main findings on the alteration of the microbiome and metabolome of cows with AD is beyond the scope of this review; therefore, we recommend consulting the work of Luo et al. [74] and Qin et al. [75].

### 3.3. Treatment

Management of cows with AD is predominantly surgical, with RDA representing a greater urgency than LDA due to the risk of developing AV [67]. The AV occurrence constitutes a surgical emergency due to the greater resulting complications [65,67]. Conservative approaches, such as the rolling technique, can be adopted in an attempt to return the abomasum to its original position and, in some cases, result in temporary resolution; however, it sometimes only represents temporary clinical improvement, making surgical intervention still necessary [61]. Notably, promoting rolling to correct LDA may predispose cows to the development of RDA or AV, which can lead to a more severe condition with a worse prognosis [29].

The timing of the intervention has direct effects on the productive and reproductive performance of animals. Late correction of AD is associated with reduced cow fertility [36]. Asgari et al. [21] also reported that LDA was associated with a reduced probability of pregnancy at first service and a 3.85-fold higher hazard of culling. This is possibly due to compromised reproductive performance, which is one of the main causes of culling dairy cows [9].

The choice of technique depends on several factors, such as preference, physical limitations, surgeon experience, treatment costs, animal value, abomasal position, and presence of a gravid uterus [26,52,67,76,77] (see Supplementary Table S2, for prognosis and outcomes). A detailed comparison between the types of techniques is beyond the scope of this review, therefore we recommend consulting the works of [9,63,66,76,77,78,79,80,81].

The choice of anesthetic block is also important. Most techniques for correcting AD are performed with the animal standing, and in these cases, local anesthesia at the incision site, with or without a paravertebral block, yields satisfactory results for digestive tract surgeries [26,82]. The use of alpha agonists, such as xylazine (0.02 mg/kg body weight, intravenous), during anesthesia can reduce stress in animals in the postoperative period of AD [83].

Restoring clinical conditions in animals with AD or AV is important for prognosis [65,80,81] (see Supplementary Table S2). Therefore, pain management is fundamental, with the use of anti-inflammatory and analgesic drugs postoperatively and, if possible, preoperatively [64,83]. Available options include flunixin meglumine-based and other anti-inflammatory drugs (e.g., ketoprofen and meloxicam) [64]. Furthermore, providing an oral solution containing electrolytes (Ca, K, Cl, Mg) to correct and restore hydro electrolytic balance and promote rumen filling is very helpful postoperatively [64,65]. Attempting to restore the balance of the rumen environment through transfaunation benefits recovering animals because it tends to improve rumen function and DMI [84]. Keeping the rumen full and functional can contribute to decreasing the recurrence of AD, especially in cases where conservative approaches have been adopted [64].

Antibiotic therapy is also essential to avoid postoperative complications such as local infections, peritonitis, and sepsis [26]. According to Mueller [63], some medications, such as erythromycin, can exert a prokinetic effect, as confirmed in humans [85]. Muller [63] recommends a dose of 10 mg/kg before surgery, which can increase the abomasal emptying rate and improve milk production in cows post-surgery; however, more robust studies in cows are required. In some cases, when animals undergo laparoscopy in surgical environments with more controlled contamination, such as operating rooms or surgical centers, antibiotics can be dispensed [77]. However, in the field, where most correction techniques are performed, the use of antibiotics is necessary because of the probability of contamination [76].

Complications after surgery can be associated with the animal’s pre-surgical clinical condition [65], quality of pre-surgical antisepsis, technique used, operator, and trans- and post-surgical care [60]. Some of the most common complications resulting from surgery are abomasal rupture, which can lead to peritonitis, hemorrhage, altered abomasal motility due to nerve damage, abomasal wall lesions, duodenal obstruction, and sepsis [26]. Alternatively, considering pregnant cows, it is possible that during attempts to correct AD, pregnancy complications such as uterine torsion and abortion may occur [29].

## 4. Abomasal Ulcers

AU has a variable prevalence among herds. In fact, the occurrence of AU in dairy cows increases considerably in the first four weeks postpartum [19]. In a review, Hund and Witteck [24] reported that some of the factors that can influence prevalence include the population examined and the diagnostic method. The authors indicated that calves make up the most susceptible category. In a study in Poland with 149 Holstein bulls evaluated in commercial slaughterhouses, Pyrek et al. [86] found a prevalence of 75.8% of erosions and 48.3% of type 1 AU.

**Figure 2:**
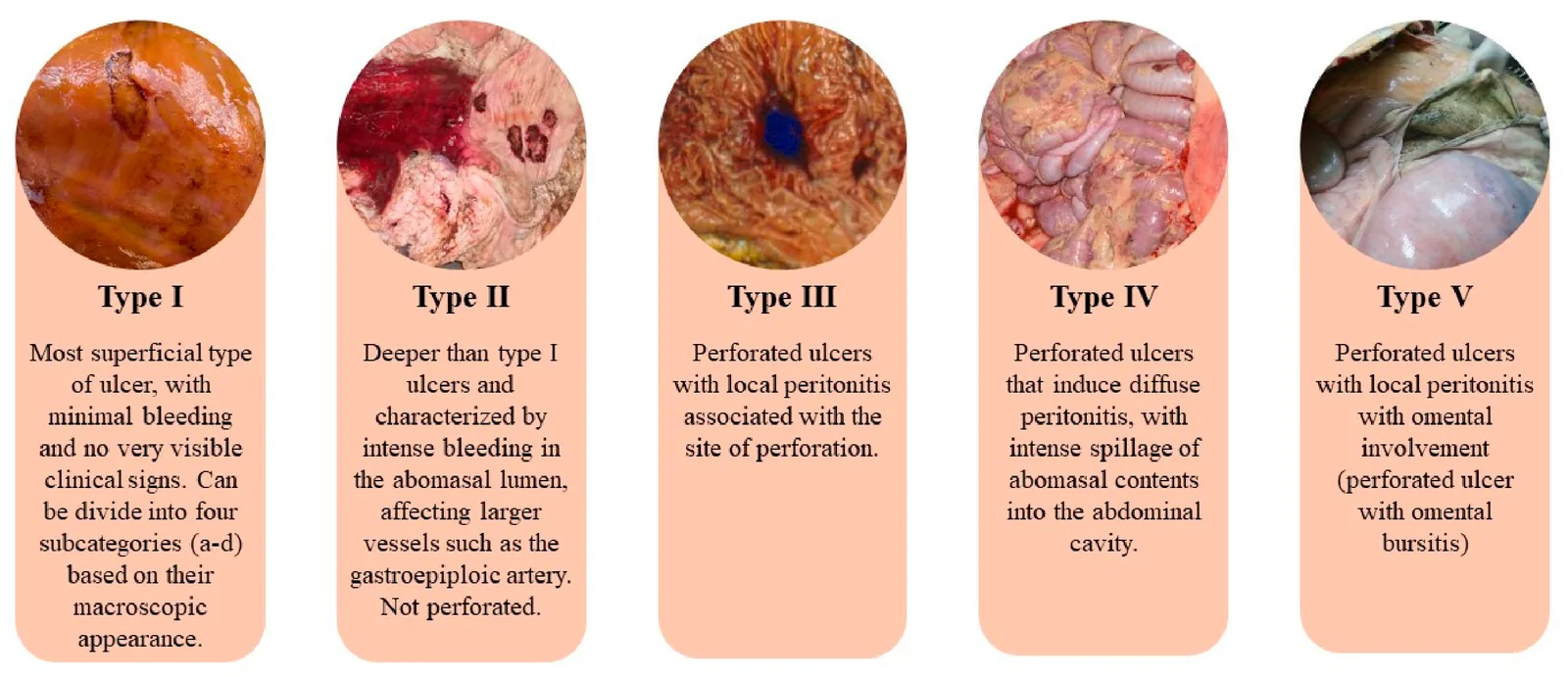
Original figure prepared by the authors showing representative gross photographs of the five types of abomasal ulcers in cattle (Types I–V), highlighting their main macroscopic characteristics.

Classifications of AU forms are important for discussing the pathophysiology, clinical signs, treatment, and prognosis [24,87,88,89,90,91,92,93,94]. The most widely adopted classification system was proposed by Smith et al. [89], who categorized AU into four types according to lesion depth, hemorrhage, and perforation. Subsequently, Braun et al. [88] refined the classification of type 1 ulcers by introducing four subcategories (1a–1d) based on their macroscopic appearance. Later, a fifth category was proposed to describe perforating ulcers associated with omental bursitis [92]. Therefore, the current classification comprises types 1–5, with type 1 further subdivided into types 1a–1d. (Figure 2).

According to Smith et al. [87] Type 1 AUs constitute the most superficial type of ulcer, with minimal bleeding and no very visible clinical signs. Braun et al. [88], in their subclassification, categorized a small erosion with tissue discoloration as a “1a” AU. Type “1b” ulcers had deeper erosions with local bleeding. Type “1c” ulcers were formed by craters with the presence of cellular debris, fibrin, or inflammatory constituents. Finally, type “1d” ulcers were most commonly located in the gastric folds, morphologically characterized by radial wrinkles converging to a central point.

Type 2 AU are characterized by intense bleeding in the abomasal lumen, affecting larger vessels such as the gastroepiploic artery, and being deeper than type 1 ulcers, although they are not perforated [90,93,94]. Type 3 AU are perforated ulcers with local peritonitis [19]. Type 4 AU are perforated ulcers that induce diffuse peritonitis, with intense spillage of abomasal contents into the abdominal cavity [92]. Finally, type 5 AU can be interpreted as a type 3 AU, but with omental involvement. Thus, it constitutes a perforated ulcer with omental bursitis [92]. The clinical aspects of each type of ulcer will be discussed further below.

### 4.1. Risk Factors

#### 4.1.1. Abomasal Displacement

The abomasum has a simple columnar epithelium. AU develops when mucosal protective mechanisms are overwhelmed by persistent chemical, mechanical, inflammatory, or infectious insults, such that the protective capacity of the mucosa is exceeded by the magnitude or duration of the injury [24]. The integrity of the abomasal mucosa depends on a dynamic balance between luminal aggressive factors, such as hydrochloric acid, pepsin, and microbial toxins, and several protective mechanisms. These include the mucus-bicarbonate layer, which limits hydrogen ion diffusion toward the epithelium, epithelial tight junctions that preserve barrier integrity, rapid epithelial restitution following superficial injury, adequate mucosal blood flow, and prostaglandin-mediated cytoprotection, which supports mucus and bicarbonate secretion while promoting tissue repair. The abomasal glandular epithelium secretes glycoproteins, particularly mucins, which form the main structural component of the mucus layer and provide protection against chemical, mechanical, and enzymatic injury [95]. Disruption of one or more of these defense mechanisms may increase mucosal permeability, facilitate hydrogen ion back-diffusion, impair tissue repair, and ultimately predispose the abomasum to ulcer formation [19,24].

As previously discussed, AD is a result of stasis or gas accumulation within the abomasum [17,23]. The emergence of AU as a consequence of AD has been discussed for some time [96]. AU appears to develop secondary in chronic cases of AD, such as LDA, where abomasal stasis contributes to prolonged retention time of abomasal contents, rich in hydrochloric acid, in the organ lumen [96]. In a study with 60 cows diagnosed with type 3 AU, Braun et al. [19] reported that 63% of the cows had some type of AD. This fact highlights the importance of early diagnosis and treatment of AD cases as a preventive strategy against the occurrence of AU.

#### 4.1.2. Stress

During the transition, cows are susceptible to various stressors, such as changes in environment, alterations in social dynamics and hierarchy, heat stress, and the occurrence of diseases, which can negatively impact the success of adaptation to the transition [20]. The combined exposure to these factors may result in a cumulative physiological burden, amplifying neuroendocrine responses such as catecholamine release and further impairing gastrointestinal motility [97]. In this context, reduced abomasal motility may arise not from a single stressor, but from the integrated effect of multiple concurrent disturbances, which collectively promote delayed gastric emptying and prolonged exposure of the abomasal mucosa to acidic content. This multifactorial stress response could therefore contribute to the development of abomasal disorders, including AD, through the interaction between altered motility, feed intake reduction, and negative energy balance.

The pathophysiology of AU due to stress in adult cows is not yet fully understood, but this association is more established in calves. AUs tend to be located more in the pyloric region in calves, while in cows they tend to be located in the fundic region [98]. The physiological response of the organism to stress could explain this association. For example, in murine models, it has been confirmed that stress significantly reduces gastric emptying by decreasing the contractile activity of the stomach [99]. Hypomotility can lead to prolonged exposure time to gastric juice [99]. In cows, stress is associated with the release and increased concentration of adrenergic hormones such as norepinephrine [97], which can inhibit the cholinergic stimulus exerted by acetylcholine [100]. Stress factors can trigger ulcerations through an integrated mechanism that includes acid hypersecretion, with decreased bicarbonate secretion and alterations in gastric contractility and emptying [101].

#### 4.1.3. Subacute Acidosis

Subacute ruminal acidosis has been associated with a drop in abomasal pH, gas accumulation, and VFAs that induce decreased motility and gastric emptying rate, factors that can also predispose to AD [55,56]. In addition, subacute ruminal acidosis is also related to increased ruminal histamine concentration, which is absorbed by the epithelium [102]. Ruminal histamine production results from the decarboxylation of the amino acid histidine, a phenomenon carried out by the microbiota, such as the bacterium *Allisonella histaminiformans*, which is seen in greater proportion when there is a drop in pH and high availability of starch, as in animals receiving diets with a higher inclusion of concentrates [103]. The role of histamine in the pathogenesis of ulcers is correlated with the effects exerted by this chemical mediator on gastric secretion, mainly via H2 receptors [104]. In lambs with AU, histamine concentrations were much higher in the abomasal contents when compared to animals without abomasal alterations [105]. The effects of histamine are associated with other disorders commonly seen in the transition, such as laminitis and mammary gland damage [104]. Thus, ulcers can be seen as consequences of this increased histamine concentration and, in turn, may be masked by the occurrence of these other disorders. A significant limitation of the study by Vatn et al. [105] is the number of animals with more severe ulcer conditions used to measure histamine in abomasal fluid (10 animals). In cows, histamine measurement in abomasal contents has not been performed; hence, future studies could be conducted to evaluate the potential of histamine as a predictive marker of AU in cattle.

#### 4.1.4. Non-Steroidal Anti-Inflammatory Drugs

The use of non-steroidal anti-inflammatory drugs increases considerably in transition cows due to their effects on controlling pyrexia, analgesia, and inflammation [106]. They are used in the treatment of various conditions such as clinical mastitis, acute puerperal metritis, and clinical ketosis [33,107]. The anti-inflammatory drugs most commonly used in cows act on the arachidonic acid cascade, selectively or non-selectively inhibiting the release of inflammatory mediators, mainly prostaglandins [107]. Some classes of prostaglandins, such as Prostaglandin E2, exert a cytoprotective effect on the abomasal mucosa, increasing mucus secretion with bicarbonate and inhibiting acid secretion [106]. Furthermore, evidence suggests that prostaglandins act on the tone of the fundic region and on the motility of the antrum and pyloric sphincter [108]. Although the use of anti-inflammatory drugs in cattle is common, there are few studies demonstrating a correlation between the administration of anti-inflammatory drugs and abomasal ulcers [18]. Walsh et al. [109] demonstrated that the use of ibuprofen (30 mg/Kg) for 10 days was able to induce ulcers in five out of eight calves, and Sasikala et al. [110] reported a case in which a type 3 AU in a bull was induced as a result of combined treatment for 7 days with flunixin meglumine (2 mg/kg) and phenylbutazone (20 mg/kg). Therefore, despite the methodological differences in these studies regarding the induction of AU or the fact that they occur with prolonged use of anti-inflammatory drugs, the use of these components should be carried out with caution because the physiological mechanisms that these anti-inflammatory drugs promote can disrupt the protective gastric balance.

#### 4.1.5. Parasitic Infections and Other Infectious Agents

Parasite species can affect cattle in various regions, including the abomasum, such as *Haemonchus* sp., *Trichostrongylus* sp., and *Ostertagia ostertagii* [111]. Infections by these parasites can result in small multifocal ulcerations of the abomasal mucosa [112]. Thus, infections with a high parasite load can cause small lesions in the abomasal mucosa, as well as diffuse mucosal nodules [86,112], which, when exposed to the acidic conditions of the abomasum, can have their diameter and depth increased. This risk factor should be considered mainly for cows kept on pasture where there is a greater risk of contamination [111].

Abomasitis due to bacterial causes has been reported in calves and adult cattle. *Clostridium perfringens* type A bacteria are most frequently seen in calves with abomasitis, but can also affect cows, causing non-perforated and perforated ulcers that can result in fibrinous peritonitis [113]. In a case–control study of 30 animals, Jelinski et al. [114] isolated *Clostridium perfringens* type A in 78.6% of the animals in the case group and in 75% of the control group, and *Escherichia coli* in more than 80% of the animals; however, none of the agents studied were associated with ulcer formation. Guarnieri et al. [115] managed to isolate *Clostridium perfringens* in six of seven abomasal cultures from calves with abomasitis. In sheep with induced acidosis inoculated with *Absidia corymbifera* spores, Chiahaya et al. [116] demonstrated that the fungus was able to induce diffuse hemorrhagic necrosis with the presence of hyphae in the lesions; however, AU were not identified, although the previously formed lesions could predispose to a future AU condition. In the case of these classes of microorganisms (bacteria and fungi), the interpretation of the isolates must be carried out cautiously, as they may indicate accidental findings and not represent a direct etiological agent. Infectious agents likely contribute to AU development by colonizing pre-existing mucosal lesions and further compromising mucosal integrity, which, when exposed to hydrochloric acid, predisposes to ulcer formation.

Knowledge regarding the role of oxidative stress in AU remains limited, particularly when compared with the evidence available for AD. In small ruminants, sheep and goats naturally infected with *Haemonchus contortus* exhibit increased malondialdehyde concentrations and reduced superoxide dismutase activity, findings consistent with enhanced oxidative stress [117]. Likewise, in humans, oxidative stress is considered an important contributor to the pathogenesis of peptic ulcers, particularly those associated with *Helicobacter pylori* infection [118]. Mechanistically, excessive production of reactive oxygen species can promote lipid peroxidation, protein oxidation, and epithelial cell damage, thereby impairing mucosal integrity and delaying tissue repair. In addition, oxidative stress may amplify local inflammatory responses, further contributing to mucosal injury [118]. Although these mechanisms have been described in other species, direct evidence supporting a similar role in bovine AU is currently lacking, and further studies are needed to clarify the contribution of oxidative stress to disease pathogenesis.

### 4.2. Diagnosis

In most cases, the diagnosis of AU is carried out through suggestive clinical presentation combined with complementary tests [89,90,91,92,93,94]. However, in some cases, differentiating them from other digestive tract conditions is practically impossible based solely on clinical presentation, requiring post-mortem identification when therapy is ineffective, and animals are euthanized or die naturally [19,92,94]. Detailed descriptions of the clinical and clinicopathological aspects of the different forms of AU have recently been reported [19,89,90,91,92,93] (see Supplementary Tables S3 and S4).

Although the clinical signs can guide the diagnosis, more than one type of AU can occur simultaneously. Therefore, in most cases, the identification and classification of lesions is only possible through exploratory laparotomy or necropsy findings [119]. Subclassifying type 1 AU as proposed by Braun et al. [88] requires a detailed inspection of the abomasal mucosa, which is quite difficult with the animal alive, although in some cases irregularities in the abomasum can be detected during exploratory laparotomy [89].

Type 2 AU is accompanied by more intense clinical and clinicopathological signs than type 1 [90]. However, other differential diagnoses can be considered, given that lymphosarcomas, some parasitoses, hemorrhagic jejunal syndrome, and other causes of bleeding in more aboral portions of the small intestine can cause it [26,49]. The plasma gastrin concentration of cows in hemorrhagic AU was double that of healthy cows (213.6 vs. 103.2 pg/mL) in the study by Ok et al. [120]. However, this study has important limitations, since the authors did not classify the types of AU, and diagnosis was made only by clinical examination or by fecal occult blood tests.

In the study by Smith et al. [121], occult blood in feces was the most reliable indicator of AU when compared to signs of pain and assessment of anemia. Diagnosis can also be made through abomasocentesis and identification of the reddish or coffee-colored abomasal fluid [122]. Nevertheless, abomasocentesis is a more invasive method that requires operator experience due to the possibility of contamination and peritonitis.

Other types of AU lead to some degree of peritonitis, so peritoneal fluid analysis can help in the diagnosis when clinical suspicion arises [91], but its occurrence should be interpreted with caution since other pathologies can cause it, such as traumatic reticuloperitonitis [15,25,26,49].

Few studies have investigated the abomasal microbiome of cows with AU. Hund et al. [123] compared the abomasal microbiota of different cattle categories (cows, calves, and bulls) presenting with AU at slaughter. Although the authors observed differences in bacterial community composition between adult animals and calves—where *Proteobacteria* and *Tenericutes* predominated in cows and bulls, whereas *Firmicutes* and *Bacteroidetes* were more abundant in calves—they did not identify specific operational taxonomic units consistently associated with ulcer formation. However, the interpretation of these findings is limited by several methodological constraints. Only type I ulcers were evaluated, and the absence of animal traceability precluded the assessment of important confounding factors, including dietary management, previous metabolic or inflammatory disorders, medication use, and production history, all of which could have influenced the abomasal microbial profile. Furthermore, because samples were collected at slaughter, the cross-sectional design did not allow determination of whether the observed microbial composition contributed to ulcer development or merely reflected lesions that had already formed. Future longitudinal studies integrating microbiome, metabolomic, and clinical data across different ulcer types are needed to characterize temporal changes in the abomasal microbiota and to identify microbial or metabolic biomarkers associated with ulcer susceptibility and progression.

To the best of our knowledge, advanced diagnostic approaches, including sensor-based technologies, machine learning, and wearable accelerometers, have not been investigated for the diagnosis of AU in cattle to the same extent as analogous technologies have been for peptic ulcer disease in humans. One promising technology is the use of a non-invasive wireless ambulatory capsule for continuous gastrointestinal pH monitoring. In calves, this approach has been successfully used to characterize abomasal pH dynamics under diarrheic conditions, demonstrating its ability to reliably detect physiologically relevant pH fluctuations [124]. However, similar applications have not yet been described in adult dairy cows to investigate abomasal pH dynamics associated with AU pathophysiology. In contrast, artificial intelligence-based diagnostic models have shown promising results in human peptic ulcer disease, with recent machine learning algorithms achieving high diagnostic accuracy [125]. Although comparable approaches have not yet been evaluated in cattle, these technologies represent a promising avenue for future AU research.

### 4.3. Treatment

The therapeutic approach for AU depends on the severity of clinical signs and the type of ulcer involved [19,89]. There are important limitations in the treatment of ulcers; milder cases, such as type 1 ulcers, tend to have greater success [89].

Type 2 ulcers can present a considerable cure rate, with responsiveness to treatment. However, the culling rate of these animals in the medium or long term can be high due to complications resulting from the original condition, the risk of recurrence, among other problems [91]. Treatment is usually based on the use of gastric protectors, antacids, and antibiotic therapy [37,89,90,122,126]. Despite the scarcity of controlled studies comparing treatment strategies for abomasal ulcers, proton pump inhibitors such as pantoprazole (1.0 mg/kg intravenously or 2.0 mg/kg subcutaneously) have been suggested to reduce gastric acid secretion in the abomasum of ruminants [126], which may contribute to improved ulcer healing under clinical conditions. This study also reported that complications may occur, such as the appearance of edema [126]. Given the cases of anemia due to intense blood loss in the feces, some animals may require blood transfusions, as well as other supportive therapies to reverse dehydration [90].

In a study with sheep, Morgado et al. [127] compared the effects of intravenous ranitidine, intravenous omeprazole, and oral omeprazole in preventing phenylbutazone-induced AU. The authors concluded that intravenous omeprazole presented adverse effects such as phlebitis and a higher incidence of abomasal lesions when compared to animals that received ranitidine. Oral omeprazole did not present adverse effects, but it was not as effective, with 50% of the animals presenting type 1 AU similar to the control group. However, it is unknown whether these same effects would be similar in cattle.

Similar therapies have been used for type 3 AU with occasional success [110]; however, the treatment described is from only one case; therefore, it is not possible to make strong inferences regarding the success of type 3 AU therapy. Most common outcomes include euthanasia or death of the animal due to the prognosis or complications [19]. Types 3, 4, and 5 AU result in some degree of peritonitis and usually result in death or euthanasia of the animals [19,91,92]. Treatment of peritonitis in ruminants is quite challenging and expensive, involves high doses of antimicrobials and anti-inflammatories, and in some cases, may require surgical intervention [15,25,128].

## 5. Knowledge Gaps and Future Perspectives

The occurrence of abomasal displacement and abomasal ulcers has been reported for over 70 years; however, there are still significant gaps in the management of these conditions. Regarding displacements, precision technologies assist in identifying suspected cases due to detected behavioral changes, but they do not replace the need for a detailed clinical examination for confirmation and differentiation from other diagnoses. Therefore, diagnostic strategies should be integrated to maximize diagnosis. Regarding clinical-hematological parameters, it is possible that there are more sensitive markers in the diagnosis of displacements, especially with the advancement of omics approaches, but more studies should be developed to elucidate them.

Compared with abomasal displacement, the immunometabolic mechanisms underlying abomasal ulcers remain incompletely characterized. Most available studies are observational, and few have directly investigated the interactions among inflammation, metabolic disturbances, mucosal integrity, and ulcer development in dairy cows. Therefore, further mechanistic and longitudinal studies are warranted. Furthermore, there is a greater tendency to neglect their occurrence, especially in cases of type 1 ulcers, which are often not diagnosed unless the animals are slaughtered or develop other types of ulcers. Additionally, there is a strong need for more robust studies with more precise and standardized methodologies for inducing ulcers, which follow the trajectory of development and can provide greater clarity regarding the pathogenesis of this disease. Finally, perhaps the main gap lies in the lack of consolidated therapeutic protocols for the treatment of abomasal ulcers when treatable. To date, no meta-analyses specifically addressing abomasal ulcers in cattle as a primary outcome have been identified in the literature, including comparisons between treatment protocols Therefore, controlled clinical trials are necessary to promote more effective therapeutic strategies.

## 6. Conclusions

This review underscores that abomasal disorders are complex conditions that can increase culling and mortality rates. The identified risk factors appear to be closely interconnected and may participate in a multifaceted network of immunometabolic alterations. However, many of these relationships are based on associative evidence, and some proposed mechanisms are inferred from studies conducted in other species rather than directly demonstrated in cattle. Therefore, further research is needed to clarify these interactions and their biological significance. From a practical perspective, veterinarians and producers should carefully evaluate the interplay among metabolic, inflammatory, and management-related factors when interpreting clinical cases and designing preventive strategies for dairy herds.

## Data Availability

Data sharing is available in the Supplementary tables.

## Supplementary Materials

The following supporting information can be downloaded at: https://www.mdpi.com/article/10.3390/ani16172706/s1. Table S1: Common clinical, clinical-pathological, and epidemiological findings in animals with left displacement abomasum (LDA), right displacement abomasum (RDA), or abomasal volvulus (AV). Table S2: Main surgical therapeutic approaches and their respective prognoses in cows with left displacement of the abomasum. Table S3: Main clinical findings and methods for confirming the diagnosis in cows with abomasal ulcers. Table S4: Main clinicopathological and ultrasonographic findings in cows with abomasal ulcers.
