# Supplementary material for: Abomasal Disorders in Dairy Cows During the Transition Period: Interconnected Risk Factors and Repercussions on Health and Culling Rate
Source: Animals (Basel). 2026 Sep 1;16(17):2706. doi: 10.3390/ani16172706 (PMC13564344; doi:10.3390/ani16172706)
Supplement: Supplementary file 1 [file animals-16-02706-s001.zip › animals-4385151-supplementary.pdf]

Supplementary Table S1. Common clinical, clinical-pathological, and epidemiological findings in animals with left displacement abomasum (LDA), right displacement abomasum (RDA), or abomasal volvulus (AV).

Reference	Number of animals, type of displacement and localization	Study objective	Common clinical and epidemiological findings	Common clinical-pathological findings
[13]	1,982 dairy cattle: LDA (1,341), RDA (338), AV (303), Swiss.	Compare clinical, laboratory, and ultrasonographic findings in cows with LDA, RDA or AV.	LDA: mild apathy, anorexia, decreased milk production, muffled rumen noises, metallic ping on the left, progressive weight loss, ballottement and simultaneous auscultation, percussion and simultaneous auscultation;<br><br>RDA: Ballottement and simultaneous auscultation, percussion and simultaneous auscultation, metallic ping on the right, right abdominal distension;<br><br>AV: more visible signs of behavioral change, initial colic, tachycardia, dehydration, enophthalmos, dark feces, absence of ruminal motility, rapid progression with worsening of the clinical signs.<br><br>87.4% of cows had at least one comorbidity. The most common comorbidity was ketosis (36.8%); Mastitis (31.5%);	In the entire population evaluated, 83% of cows had hypokalemia, 78% had hypocalcemia, 67% had a base deficit, 67% had an increase in rumen chloride, 50% had hemoconcentration, 45% had hypochloremia, 40% had increased bicarbonate concentration in the blood and 31% had azotemia.<br><br>LDA: Increased bilirubin (89%), gamma glutamyl transferase (52%) Ketonuria (58%), Hemoconcentration (45%).<br><br>RDA: Increased aspartate transaminase (85%), L-Lactate (79%); gamma glutamyl transferase (79%);

			Metritis/endometritis (27%); Hepatic Lipidosis (21.7%).	Hemoconcentration (61%); Rumen Chloride (51%).<br><br>AV: Increased gamma glutamyl transferase (84%); L-Lactate (79%); inorganic phosphorus (68%) Hemoconcentration (63%); urea (61%)
[64]	662 dairy cattle LDA, Germany	Evaluate treatment, concomitant diseases, and prognosis.	38.4% presented with metritis/endometritis; 28% of the cows had concomitant subclinical ketosis, which influenced the survival rate ($p<0.05$). Other identified conditions included septicemia, mastitis, clinical ketosis, hypocalcemia, and hoof lesions, with varying prevalence between 12.7%-22.4%, but without influencing the survival rate.	In cows that died, there was an increase in glucose, L-Lactate, total protein, aspartate aminotransferase, gamma glutamyl transferase, P. There was a reduction in base excess, Na, K, Cl.
[65]	234 dairy cattle (182 RDA and 52 AV) Germany	Identify clinical and laboratory predictors of the outcome.	Non-survivors (41 cows) presented with severe dehydration, marked tachycardia (72%), severely decreased skin tent (63%), anorexia (76%), ruminal hypomotility (45%) or atony (35%), and abomasum accessible at rectal examination (65%); animals unable to stand had a fatal outcome; survivors had milder clinical signs.	In non-surviving cows, there was a reduction in Na, K, and Cl, and an increase in phosphorus and lactate, hypoproteinemia; metabolic acidosis or alkalosis, depending on the degree of obstruction; AV obstruction was frequently associated with severe dilation and torsion with vascular compromise.

[27]	135 cases (127 LDA, 9 RDA) in 39 heards (6,454 cows, 2,987 lactating cows) - Brazil	Retrospective study to determine prevalence and epidemiological characteristics.	Predominantly occurs in multiparous Holstein cows during the transition period (first 4 weeks postpartum, 84%); higher risk in winter; clinical signs similar to those described in the literature (metallic ping, anorexia, reduced milk production).	The authors did not detail individual laboratory tests.
[66]	Herd with 400 multiparous Holstein cattle; 7 with LDA, Iran	A case-control study whose objective was to compare and evaluate biomarkers of oxidative stress and attempt to find predictive metabolites.	Apathy, reduced appetite and milk production >25%, metallic drip during percussion and auscultation of the left abdominal regions. Ultrasound examination confirmed the diagnosis by visualizing reverberation artifacts and was confirmed by exploratory laparotomy.	Cows with LDA had increased urea, blood urea nitrogen, gamma-glutamyl transferase, glucose, cholesterol, triglycerides, β-hydroxybutyrate, aspartate transaminase, serum amyloid A, malondialdehyde, and reduced total antioxidant capacity compared to healthy cows, indicating greater oxidative stress and lower antioxidant capacity.

Supplementary Table S2 - Main surgical therapeutic approaches and their respective prognoses in cows with left displacement of the abomasum.

Reference	Animals evaluated	Study objective	Key results	There was an informed prognosis, and how was it assessed?	Duration of monitoring
[78]	87	To evaluate the reproductive performance and productive longevity of cows treated with omentopexy versus pyloro-omentopexy	Omentopexy resulted in a 14% recurrence rate, while pyloro-omentopexy had no recurrence. Slaughter rates and longevity were similar to those of unaffected cows.	Good longevity prognosis. Better prognosis for non-recurrence with pyloromentopexy. Prognoses were evaluated through a review of herd records for longevity, reproductive performance, and LDA recurrence.	Up to 4 years after the last surgery.
[79]	129	Compare the culling rate of cows treated with right omentopexy, alternating suture, or a conservative approach (without surgery)	Using the Cox regression model, the risk of culling was, respectively, 9, 10, and 37-fold higher than in healthy cows.	Prognoses were not assessed, only the risk of culling,	In the first 300 days of lactation

[80]	30	To compare the clinical and metabolic outcomes of one-step laparoscopic abomasopexy with abomasopexy via the right paralumbar fossa.	Laparotomy was faster. Both techniques restored abomasal flow and food intake.	Similar effectiveness in restoring digestive function and consumption. Prognoses assessed through Measurement of clinical, metabolic, and electrolyte parameters (e.g., pH, bicarbonate).	Up to 72 hours after surgery.
[81]	89	To compare the survival rate and biochemical profile of cows treated with laparoscopic abomasopexy (LS) vs. laparotomic omentopexy (LT).	The survival rate was similar (loss of ~11%). The LS group showed more favorable biochemical dynamics in the postoperative period (Total protein, calcium, magnesium, and albumin).	Assessment of survival rate and biochemical profile (blood collection). The survival rate was similar (loss of ~11%).	30 days after surgery.

[77]	15 pregnant cows.	To investigate the postoperative performance of one-step laparoscopic abomasopexy in cattle in late pregnancy.	Satisfactory results in 100% of the cows. There were no complications before calving.	Observation of postoperative complications, recording of reproductive performance, and calculation of survival rate. Satisfactory, with good reproductive performance. One-year survival rate of 60%. The average culling interval of animals is 415 days after surgery. In most cases, the cause of death is not mentioned.	One year (for survival rate).
[12]	126	Comparing two surgical techniques (laparoscopy vs. laparotomy) regarding the return of milk production.	The return of milk production did not differ significantly between the groups.	Comparison of milk production (milking records). There is no significant difference between the techniques.	Before surgery and at 8, 15, and 30 days after surgery.

[52]	5 pregnant cows.	To evaluate the clinical utility of the surgical tilting pin suture technique (s-TPS) in pregnant cows with LDA.	s-TPS technique is a practical and effective option. Short surgical time.	Assessment of postoperative survival. 100% survival rate (5/5).	More than 2 years post-surgery.

Supplementary Table S3 - Main clinical findings and methods for confirming the diagnosis in cows with abomasal ulcers.

Reference	Type of Ulcer	Animals evaluated	Description of the main clinical findings	How was the diagnosis confirmed?
[89]	Type 1	94	Compromised general health status (94%); partial or complete anorexia (93%); congested episcleral vessels (89%); decreased skin surface temperature (76%); decreased or absent fecal production (72%); abdominal guarding (59%); tachypnea (56%); ruminal atony (53%); positive percussion, auscultation, and ballottement on the right side (53%); tachycardia (50%); positive foreign body tests (47%); abdominal pain (39%); fever (35%).	91.5% post-mortem and 8.5% through laparotomy by visually observing the abomasal wall or palpating it.
[90]	Type 2	145	Depressed behavior (100%); partial or complete anorexia (93%); ruminal atony (91%); tachycardia (90%); melena (80%); prolonged capillary refill time (71%); pale mucous membranes (69%); tachypnea (62%); hypothermia (58%); abdominal guarding (34%); abdominal pain (30%).	86% of the cows were diagnosed immediately after the initial examination, the remaining 14% only after signs of gastrointestinal bleeding. 36.5% had post-mortem confirmation due to euthanasia or natural death.

[19]	Type 3	60	Partial or complete anorexia (98%); obtuse/depressed behavior (95%); decreased skin surface temperature (78%); congested episcleral vessels (73%); reduced fecal production (65%); abdominal guarding (61%); tachypnea (58%); fever (58%); tachycardia (55%); ruminal atony (49%); positive foreign body test (45%); ruminal hypomotility (41%); hypothermia (20%); bruxism (18%); arched back (13%); melena (10%).	60% post-mortem and 40% via exploratory laparotomy.
[91]	Type 4	87	Compromised health status and partial/complete anorexia (100%); abdominal guarding (81%), congested episcleral vessels (77%); ruminal atony (73%); tachycardia (68%); tachypnea (65%); positive foreign body tests (58%); decreased skin temperature (53%); fever (49%); negative intra-abdominal pressure (39%); presence of large fibers in feces (35%); arched back (28%).	87% of the cows showed signs suggestive of peritonitis on ultrasound (for example: generalized echogenic lesions and free fluid on the abdomen; echogenic changes with or without fluid inclusions on the reticulum). 100% of the animals were confirmed *post-mortem*.
[92]	Type 5	14	Partial or complete anorexia (100%); abdominal guarding (100%); obtuse behavior (93%); congested scleral vessels (79%); tachypnea (71%); ruminal atony (64%); decreased fecal production (64%); reduced skin surface temperature (64%); fever (46%).	100% *post mortem.*

Supplementary Table S4 - Main clinicopathological and ultrasonographic findings in cows with abomasal ulcers

Reference	Type of Ulcer	Animals evaluated	Clinicopathological findings	Ultrasonographic findings
[89]	Type 1	94	Hypokalemia (68%); increased base excess (60%); increased pCO2 (52%); azotemia (51%); increased ruminal chloride (48%); hemoconcentration (45%); hyperfibrinogenemia (36%); leukopenia (26%).	Nonspecific signs, with no evidence of ulceration. Only some changes, such as abomasal dilation (26%); free fluid in the reticular region (19%); reduced intestinal motility (18%).
[90]	Type 2	145	Azotemia (89%); decreased hematocrit (82%); hypokalemia (81%); hypoproteinemia (74%); metabolic acidosis (61%); hyperbilirubinemia (25%); hypoalbuminemia (15%).	Ultrasound was performed on only 26% of the animals (38 cows). Of these, 8 presented with abomasal dilation, 5 cows were displaced to the left, 3 displaced to the right, and 2 had clots in the intestinal lumen.
[19]	Type 3	60	Hypokalemia (75%); increased base excess (50%); reduced glutaraldehyde test time (46%); increased pCO2 (46%); reduced blood pH/acidosis (45%); hyperfibrinogenemia (43%); hemoconcentration (35%); azotemia (35%); hypochloremia (34%); altered peritoneal fluid (30%); leukocytosis (25%).	Abomasum displaced to the left (59%); echogenic changes in the reticulum with or without fluid inclusions (40%); reticular atony (36%). Free fluid in the abomasal region (22%).

[91]	Type 4	87	Changes in peritoneal fluid (increased specific gravity and/or increased protein concentration, green discoloration) (98%); hypokalemia (72%); hemoconcentration (69%); azotemia (56%); aciduria (56%); metabolic acidosis (49%); hyperfibrinogenemia (45%); hematuria (44%); leukopenia (35%); hypoproteinemia (29%).	Evidence of local or generalized peritonitis (87%); reticular atony (41%) and echogenic changes in the reticulum with or without fluid inclusions (46%). Predominance of generalized echogenic lesions in the abdomen (41%).
[92]	Type 5	14	Hypokalemia (71%); hemoconcentration (57%); metabolic acidosis (57%); azotemia (43%); hyperfibrinogenemia (36%); leukopenia (36%); altered peritoneal fluid (29%).	Echogenic changes in the reticular formation with or without fluid inclusions (60%); generalized free fluid in the abdomen (50%); reticular atony (40%); abnormal reticular contour (40%).
